# Effects of different sedation regimens on delirium in ICU patients

**DOI:** 10.3389/fneur.2026.1745283

**Published:** 2026-05-15

**Authors:** Fei Liu, Jie Yao, Liqian Zhang, Naikun Gao, Zhifei Qiao, Lei Wang, Chunyan Liu, Fulong Li

**Affiliations:** 1 Intensive Care Unit, The First Affiliated Hospital of Hebei North University, Zhangjiakou, Hebei, China; 2 Department of Anesthesiology, The First Affiliated Hospital of Hebei North University, Zhangjiakou, Hebei, China

**Keywords:** delirium, dexmedetomidine, intensive care unit, midazolam, remimazolam

## Abstract

**Background:**

Delirium is common among ICU patients and worsens outcomes. The comparative effectiveness of sedatives remains uncertain.

**Methods:**

We conducted a single-center, prospective cohort study using a prespecified 48-h landmark. Adults (≥18 years) with an expected ICU stay ≥24 h and continuous intravenous sedation ≥6 h were enrolled. The predominant sedative used during the first 48 h (dexmedetomidine, midazolam, or remimazolam) was recorded. Predominance was defined *a priori* as the agent with the greatest infusion time share, with ties broken first by standardized cumulative dose and then by earliest start time/longest continuous block. The primary outcome was incident delirium from hour 48 to ICU day 7 or discharge, as determined using standardized, sedation-gated CAM-ICU assessments. Secondary outcomes included time-in-target sedation, ventilator-free days, and prespecified hemodynamic adverse events. We used multinomial propensity-score overlap weighting and weighted log-link models to estimate risk ratios (RRs) with robust standard errors. A time-varying exposure analysis from ICU admission assessed robustness.

**Results:**

Of the 506 patients who were screened, 426 were included in the landmark cohort (dexmedetomidine = 162, midazolam = 192, and remimazolam = 72). Incident delirium occurred in 108 (25.4%) of the 426 patients. After overlap weighting, dexmedetomidine was associated with a lower risk of delirium versus midazolam (RR 0.70, 95% CI 0.52–0.92) and a similar, although not significant, pattern versus remimazolam (RR 0.74, 95% CI 0.52–1.03). Dexmedetomidine had a higher time-in-target sedation and more ventilator-free days, with more bradycardia, but no apparent increase in hypotension or hypertension. Mortality did not differ. The findings were directionally consistent in the time-varying analysis (HR 0.76, 95% CI 0.58–0.99).

**Conclusion:**

In this prospective cohort, dexmedetomidine-predominant sedation was associated with a lower risk of post-landmark delirium and better sedation quality than midazolam, at the cost of more bradycardia and without differences in mortality. These findings warrant multicenter confirmation and randomized evaluation.

## Introduction

Delirium is a prevalent and severe complication in adult ICU patients, particularly those on mechanical ventilation, and is associated with an increased duration of ventilation, length of stay (LOS), and mortality. Across studies, delirium has been found to affect approximately 20–50% of adult ICU patients and up to 60–80% of mechanically ventilated adults, with important downstream consequences including long-term cognitive impairment among survivors (1, 2). The choice of sedatives and the depth of sedation are modifiable risk factors that can influence the incidence of delirium. Dexmedetomidine, an alpha-2 adrenergic agonist, has been shown to reduce the incidence of delirium compared to other sedatives, such as benzodiazepines and propofol. Studies indicate that dexmedetomidine not only decreases the occurrence of delirium but also improves patient outcomes by reducing ICU LOS and preserving cognitive function without causing significant respiratory depression (3–5). In contrast, benzodiazepines have been associated with higher delirium rates and longer ICU stays (3, 6). However, dexmedetomidine can lead to bradycardia and hypotension (6). The evidence supporting dexmedetomidine’s efficacy is robust, with multiple studies and meta-analyses confirming its benefits in reducing delirium incidence and duration, although some studies have suggested that its use is more prevalent in severely ill patients (6, 7). Nevertheless, existing trials and meta-analyses often have restrictive eligibility criteria, heterogeneous delirium surveillance, and limited comparators, leaving uncertainty about the comparative risk of delirium among contemporary sedatives in routine ICU practice (3, 8). Non-pharmacological interventions remain crucial in delirium management, emphasizing the importance of a comprehensive approach that includes both pharmacological and non-pharmacological strategies (1, 2). While the debate between dexmedetomidine and benzodiazepines continues, the current evidence leans toward dexmedetomidine as a more effective option for reducing delirium in ICU settings (3, 9).

Exploring remimazolam’s impact on postoperative delirium and cognitive function reveals a complex landscape, particularly in comparison to non-benzodiazepine strategies. A systematic review indicated that remimazolam does not significantly increase the incidence of delirium compared to other sedatives (10). A trial involving older adults undergoing orthopedic surgery found that delirium was observed at comparable rates with remimazolam (15.6%) and propofol (12.4%) (11). Furthermore, remimazolam demonstrated superior short-term cognitive function on the seventh postoperative day (10). Remimazolam is a benzodiazepine, but its ultra-short-acting, esterase-metabolized profile may reduce drug accumulation and facilitate lighter, more arousable sedation than that of longer-acting benzodiazepines, such as midazolam, potentially translating to a different risk of delirium in ICU sedation practices (12, 13). Despite its favorable pharmacological profile, which includes rapid onset and minimal cardiorespiratory depression, the need for more head-to-head comparisons and standardized delirium surveillance is critical to fully understand its role in clinical practice (12, 13). Importantly, the majority of available remimazolam delirium data are from the perioperative period with a short follow-up and limited ICU-specific comparators, leaving the comparative risk of delirium during prolonged ICU sedation uncertain (10–13).

Our primary objective was to compare the risk of incident ICU delirium occurring after a prespecified 48-h landmark (from hour 48 through ICU day 7 or ICU discharge) among adults predominantly sedated with dexmedetomidine, midazolam, or remimazolam during the initial 48 h of ICU care. This landmark approach was chosen to align sedative exposure classification with subsequent delirium risk timing and to mitigate reverse causation and immortal-time bias when early delirium may influence sedative choice (14). The secondary objectives were to compare sedation quality (time-in-target sedation), ventilator-free days, ICU length of stay, 28-day mortality rates, and prespecified hemodynamic adverse events (bradycardia, hypotension, and hypertension) across sedative groups.

## Methods

### Study design and setting

**Figure 1:**
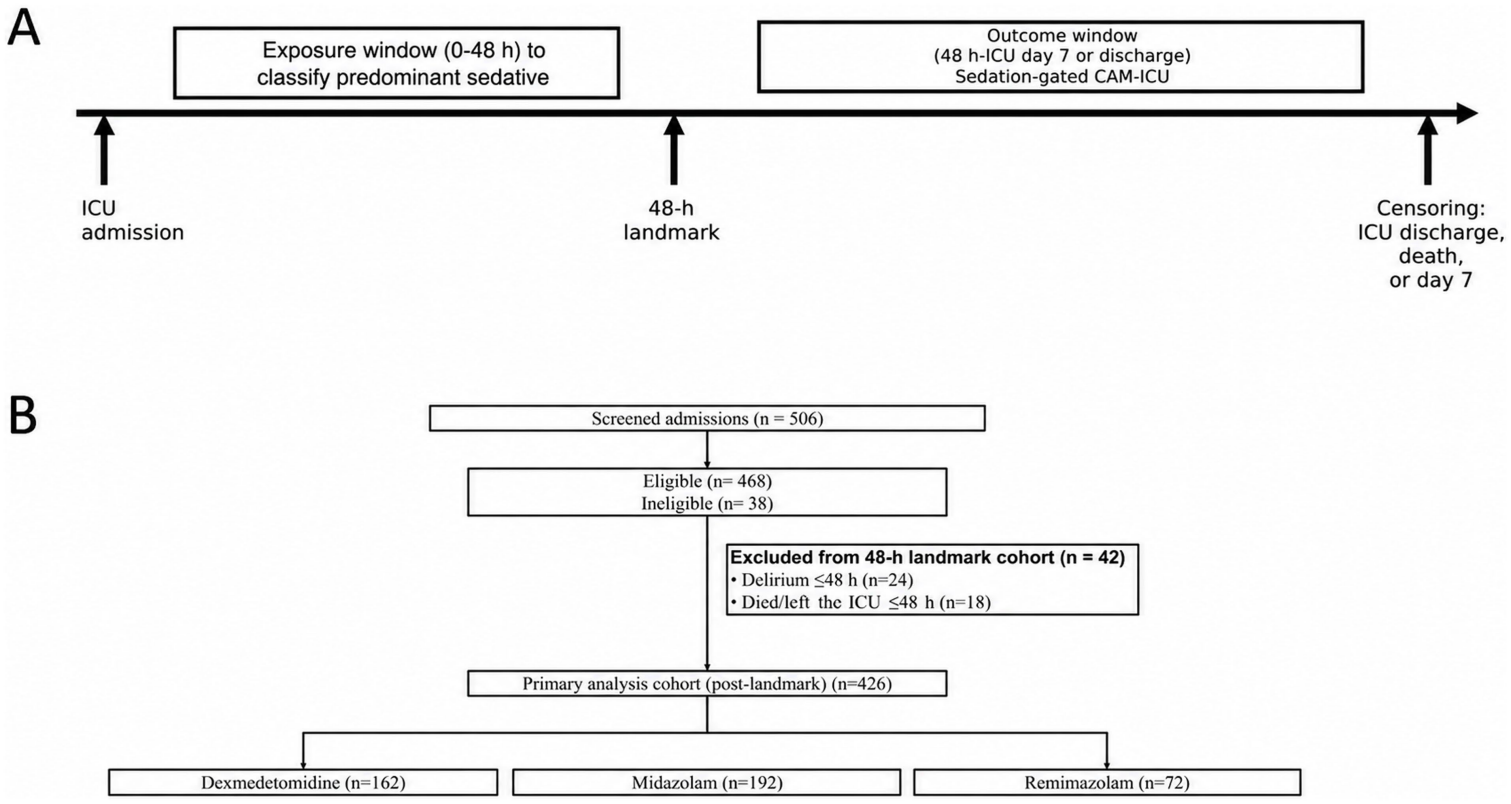
Study design timeline and flow diagram. **(A)** Exposure window (0–48 h) used to classify predominant sedative; incident delirium assessed from hour 48 to ICU day 7 or discharge using sedation-gated CAM-ICU; censoring at ICU discharge, death, or day 7. **(B)** Flow of screened admissions (Aug 2023–Apr 2025) through eligibility and landmark exclusions (delirium ≤48 h; death/ICU discharge ≤48 h), and the post-landmark analysis cohort with group allocation.

We conducted a single-center, prospective, observational cohort study at The First Affiliated Hospital of Hebei North University from August 2023 to April 2025, following a prespecified protocol. The protocol conformed to the Declaration of Helsinki and was approved by the ethics committee of The First Affiliated Hospital of Hebei North University. All participants provided written informed consent prior to any study procedures. To align sedative regimen classification with outcome risk time and minimize immortal-time bias (14), the primary analysis used a 48-h landmark with follow-up from hour 48 through ICU day 7 or ICU discharge (Figure 1A).

### Participants

Consecutive adults aged ≥18 years were eligible if their expected ICU stay was ≥24 h and they received continuous IV sedation for ≥6 h within the first 48 h after ICU admission; for the primary analysis, we excluded patients who developed delirium within 48 h or who died/left the ICU before hour 48, while retaining these individuals for sensitivity analyses that did not rely on the landmark restriction to ensure adequate exposure opportunity and correct temporal ordering of exposure and outcome.

### Exposure definition

We defined the predominant sedative during hours 0–48 as the agent accounting for the largest share of continuous infusion time within that window. All three study sedatives could be used sequentially or in combination; predominance did not require > 50% exposure and was assigned to reflect the agent most commonly used during the window. Ties were resolved using (i) a standardized cumulative dose (drug-class–specific units harmonized *a priori*) and, if still tied, (ii) the earliest start with the longest continuous block. To describe exposure stability, we summarized the proportion of infusion time accounted for by the predominant agent and the frequency of mixed/sequential use of ≥2 study sedatives during hours 0–48. Exposure status was fixed at the 48-h landmark for the primary analysis. Post-landmark switching was addressed in time-varying sensitivity analyses.

### Outcomes and measurements

The primary outcome was incident delirium occurring after the 48-h landmark through ICU day 7 or discharge. Bedside nurses trained in standardized procedures performed CAM-ICU assessments (15) at least twice daily (once per nursing shift) and after major sedation changes, with evaluations gated to an arousal level adequate for testing [Riker Sedation Agitation Scale (SAS) 3–5] to avoid false negatives under deep sedation. When arousal was insufficient (SAS < 3), CAM-ICU was recorded as “unable to assess” and repeated at the next scheduled assessment; we summarized unassessable periods and overall assessment completeness by sedative group. Sedation depth and quality were measured with the Riker SAS every 2–4 h (16) with a prespecified target of SAS 3–4, and time in target sedation was defined as the proportion of assessments within this range over the post-landmark window. Safety endpoints included bradycardia requiring intervention, prespecified as a heart rate <50 bpm with atropine administration, temporary pacing, vasopressor escalation for bradycardia, or sedative down-titration/hold attributed to bradycardia. Additional hemodynamic safety endpoints were hypotension requiring intervention (new initiation or dose escalation of vasopressors or a fluid bolus for systolic blood pressure <90 mmHg or a mean arterial pressure <65 mmHg, attributed by the treating team to sedation) and hypertension requiring treatment (intravenous antihypertensive administration or sedative adjustment for a systolic blood pressure ≥180 mmHg, attributed to sedation). Patient-centered outcomes included ventilator-free days (VFDs) up to day 28, ICU length of stay, and 28-day mortality rates.

### Covariates

We recorded baseline and early ICU variables that were chosen *a priori* for their potential to influence both sedative selection and risk of delirium, including demographics, admission features, illness-severity markers (shock/vasopressor use, mechanical ventilation at baseline, sepsis or systemic infection, neurological injury or baseline cognitive impairment), organ dysfunction (renal or hepatic), and concurrent psychoactive/analgesic medications, and we used these covariates in the treatment assignment model to address confounding by indication.

### Analysis of population and bias mitigation

Our analysis defined “Time zero” as the 48-h landmark, and only patients alive and without delirium at hour 48 entered the primary cohort to preempt immortal-time and reverse-causation bias; unadjusted group incidences and risk ratios are reported for transparency, but primary causal inference relied on covariate-adjusted models after propensity-score weighting in an overlap population reflecting areas of clinical equipoise.

### Statistical analysis

We estimated a multinomial propensity score for the three sedative strategies using prespecified covariates and applied overlap weighting to target the average treatment effect in the overlapping (equipoise) population. Balance was assessed via standardized mean differences (target absolute SMD < 0.10) and effective sample size summaries post-weighting. The primary effect measure for incident delirium was the risk ratio (RR) from a generalized linear model with a log link and robust (sandwich) standard errors under the overlap weights, with modified Poisson with robust variance used if log-binomial models failed to converge. Adjusted absolute risk differences with 95% confidence intervals (CIs) were reported for the primary contrast. Secondary outcomes used the same weighting with mean differences and robust variance for continuous outcomes (time-in-target sedation in percentage points and VFDs in days) and RRs for binary safety. Hypotension and hypertension were analyzed as additional (exploratory) safety endpoints and were presented with nominal *p*-values. Two-sided p-values were reported, and within prespecified families, we controlled multiplicity using the Benjamini–Hochberg false discovery rate (FDR): two primary pairwise contrasts (dexmedetomidine vs. midazolam; dexmedetomidine vs. remimazolam) and three secondary endpoints (time-in-target, bradycardia, and VFDs), with q-values shown alongside nominal *p*-values. Unadjusted estimates used risk ratios with Fisher’s exact test p-values for group comparisons. Missing covariates were handled via multiple imputation by chained equations, and outcomes were not imputed. No formal *a priori* sample size calculation was performed for this observational cohort; the study size was determined by consecutive enrollment during the study period, and precision was conveyed by confidence intervals (with wider intervals anticipated for the smaller remimazolam group).

*Sensitivity analysis*: Because sedative exposure can change after 48 h, we conducted a prespecified time-to-event sensitivity analysis from ICU admission in which sedative class was treated as a time-varying covariate, updating exposure whenever the predominant continuous sedative changed; follow-up continued until the first occurrence of delirium, ICU discharge, death, or ICU day 7, whichever came first. Additional robustness checks for competing risks (ICU discharge and death before delirium as competing events using Fine–Gray subdistribution models) and alternative propensity-score specifications were prespecified. We additionally performed (1) an as-treated landmark analysis starting at hour 48 and censoring at the first switch away from the predominant sedative (or addition of another continuous sedative infusion >6 h), and (2) a worst-case analysis reclassifying patients with ≥2 consecutive “unable to assess” CAM-ICU evaluations (SAS < 3) as having delirium, to evaluate potential bias from differential delirium assessability under deep sedation.

## Results

### Patient enrollment and baseline characteristics

Of the 506 ICU admissions that were screened, 468 met the eligibility criteria, and 42 were excluded from the 48-h landmark cohort for early delirium (*n* = 24) or death/ICU discharge ≤48 h (*n* = 18), yielding 426 patients for the primary analysis, which were distributed as follows: dexmedetomidine (*n* = 162, 38.0%), midazolam (*n* = 192, 45.1%), and remimazolam (*n* = 72, 16.9%) (Figure 1B).

Among the post-landmark cohort, baseline features were broadly comparable across groups (Table **1**). The median age was clustered at 65–66 years, and the proportion of female patients was similar. Illness acuity and support needs were closely aligned: mechanical ventilation at enrollment occurred in 82.1, 83.9, and 80.6% of patients in the dexmedetomidine, midazolam, and remimazolam groups, respectively; vasopressor use within 24 h occurred in 48.1, 50.0, and 45.8% of patients, respectively; and SOFA medians were 8 [6–10], 8 [6–11], and 7 [5–10], respectively. Similarly, comorbid and presenting conditions were balanced, including sepsis at admission (42.0, 43.2, and 41.7%), baseline cognitive impairment/dementia (6.8, 7.8, and 6.9%), renal dysfunction (19.1, 20.8, and 18.1%), and hepatic dysfunction (11.1, 12.0, and 9.7%). Early concomitant therapies were also common and similar: opioid co-analgesia was used in 74.1, 75.0, and 73.6% of patients, respectively, and antipsychotic exposure was present in 11.7, 13.0, and 12.5% of cases, respectively—supporting clinical equipoise at baseline and the suitability of overlap-weighting to address residual imbalance in comparative analyses (Table 1).

**Table 1:** Baseline and exposure-window characteristics by sedative group.

Characteristic	Dexmedetomidine (*n* = 162)	Midazolam (*n* = 192)	Remimazolam (*n* = 72)
Age (years), median [IQR]	65 [54–74]	66 [55–75]	65 [53–74]
Female, *n* (%)	62 (38.3%)	69 (35.9%)	27 (37.5%)
Mechanical ventilation at enrollment, *n* (%)	133 (82.1%)	161 (83.9%)	58 (80.6%)
Vasopressors within 24 h, *n* (%)	78 (48.1%)	96 (50.0%)	33 (45.8%)
SOFA score at enrollment, median [IQR]	8 [6–10]	8 [6–11]	7 [5–10]
Sepsis on admission, *n* (%)	68 (42.0%)	83 (43.2%)	30 (41.7%)
Baseline cognitive impairment/dementia, *n* (%)	11 (6.8%)	15 (7.8%)	5 (6.9%)
Alcohol use disorder, *n* (%)	15 (9.3%)	19 (9.9%)	6 (8.3%)
Renal dysfunction, *n* (%)	31 (19.1%)	40 (20.8%)	13 (18.1%)
Hepatic dysfunction, *n* (%)	18 (11.1%)	23 (12.0%)	7 (9.7%)
Concomitant opioids, *n* (%)	120 (74.1%)	144 (75.0%)	53 (73.6%)
Antipsychotic exposure, *n* (%)	19 (11.7%)	25 (13.0%)	9 (12.5%)
Predominant sedative share of infusion time during hours 0–48 (%), median [IQR]	84 [74–92]	86 [78–93]	81 [70–89]
Mixed/sequential use of ≥2 study sedatives during hours 0–48, n (%)	54 (33.3%)	61 (31.8%)	30 (41.7%)

### Exposure stability and delirium assessment completeness

During hours 0–48, the predominant agent accounted for a median of 84% [74–92], 86% [78–93], and 81% [70–89] of infusion time in the dexmedetomidine, midazolam, and remimazolam groups, respectively, and mixed/sequential use of ≥2 study sedatives occurred in 54 (33.3%), 61 (31.8%), and 30 (41.7%) patients, respectively (Table **1**). From hour 48 to ICU day 7/discharge, 5,140 CAM-ICU assessments were attempted (median 12 [10–14] per patient), of which 715 (13.9%) were recorded as “unable to assess” due to deep sedation (SAS < 3). There were 39 patients with ≥1 unassessable assessment (24.1%) in the dexmedetomidine group, 63 (32.8%) in the midazolam group, and 25 (34.7%) in the remimazolam group (Table 2).

**Table 2:** Delirium assessment completeness and deep sedation (post-landmark).

Assessment metric	Dexmedetomidine (*n* = 162)	Midazolam (*n* = 192)	Remimazolam (*n* = 72)	Overall (*n* = 426)
CAM-ICU assessments attempted, *n*	1,950	2,340	850	5,140
CAM-ICU assessments per patient, median [IQR]	12 [10–14]	12 [10–15]	11 [9–14]	12 [10–14]
Unassessable (SAS<3), *n* (% of attempts)	210 (10.8%)	360 (15.4%)	145 (17.1%)	715 (13.9%)
Patients with ≥1 unassessable assessment, *n* (%)	39 (24.1%)	63 (32.8%)	25 (34.7%)	127 (29.8%)
Patients with ≥2 consecutive unassessable assessments, *n* (%)	18 (11.1%)	31 (16.1%)	13 (18.1%)	62 (14.6%)

### Primary outcome: incident delirium after the 48-h landmark

Between hour 48 and ICU day 7 or discharge, 108 (25.4%) patients developed incident delirium, with group incidences of 33 (20.4%) for dexmedetomidine, 55 (28.6%) for midazolam, and 20 (27.8%) for remimazolam (Table 3). The unadjusted risk ratios versus midazolam were 0.71 (95% CI, 0.49–1.04; *p* = 0.084) for dexmedetomidine and 0.97 (95% CI, 0.63–1.50; *p* = 1.000) for remimazolam, and the corresponding absolute risk differences were −8.3% (95% CI, −17.2 to 0.6%) and −0.9% (95% CI, −13.0 to 11.3%), respectively (Table 3).

**Table 3:** Primary event rates by sedative group (unadjusted).

Group	Delirium, n/N (%)	Unadjusted RR vs. Mid (95% CI)	Absolute risk difference vs. Mid, % (95% CI)	p (Fisher)
Dexmedetomidine	33/162 (20.4%)	0.71 (0.49–1.04)	−8.3 (−17.2 to +0.6)	0.084
Midazolam	55/192 (28.6%)	—	—	—
Remimazolam	20/72 (27.8%)	0.97 (0.63–1.50)	−0.9 (−13.0 to +11.3)	1.000

**Figure 2:**
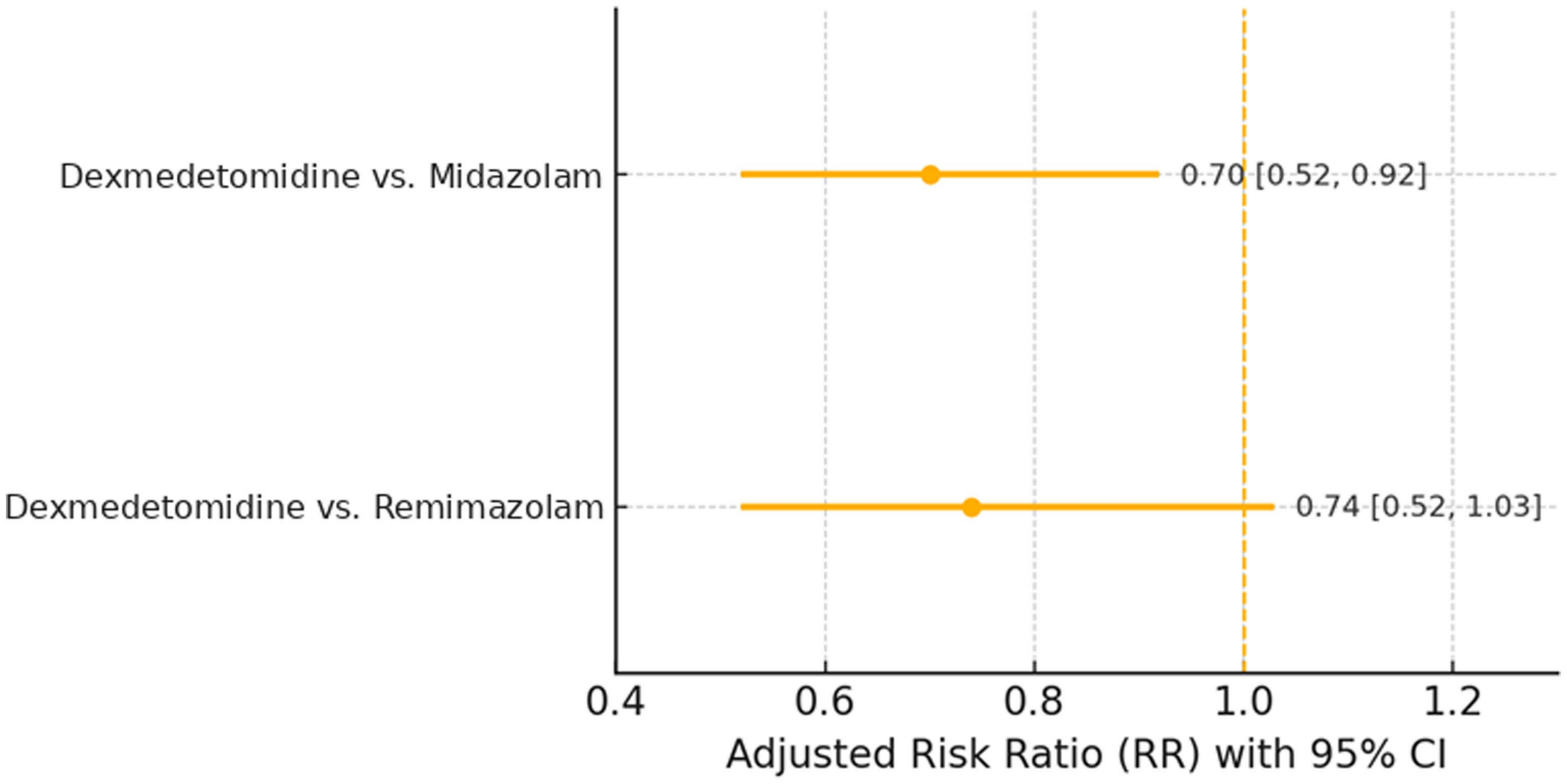
Incident delirium (post-landmark), overlap-weighted risk ratios. Adjusted RRs with 95% CIs for dexmedetomidine vs. midazolam (0.70 [0.52–0.92]) and dexmedetomidine vs. remimazolam (0.74 [0.52–1.03]); vertical line at RR = 1.0.

After applying multinomial propensity-score overlap weighting and log-link modeling, dexmedetomidine was associated with a significantly lower risk of delirium than midazolam (adjusted RR 0.70, 95% CI 0.52–0.92; adjusted absolute risk difference −8.7, 95% CI − 15.9 to −1.7%; *p* = 0.010; FDR q = 0.020), whereas the comparison with remimazolam was directionally similar but not statistically significant (adjusted RR 0.74, 95% CI 0.52–1.03; *p* = 0.080; FDR q = 0.080) (Figure 2 and Table 4).

**Table 4:** Primary comparative effectiveness (overlap-weighted).

Contrast (post-landmark)	Adjusted RR (95% CI)	Adjusted ARD, % (95% CI)	*p*	FDR q
Dexmedetomidine vs. Midazolam	0.70 (0.52–0.92)	−8.7 (−15.9 to −1.7)	0.010	0.020
Dexmedetomidine vs. Remimazolam	0.74 (0.52–1.03)	—	0.080	0.080

### Secondary outcomes and safety

**Figure 3:**
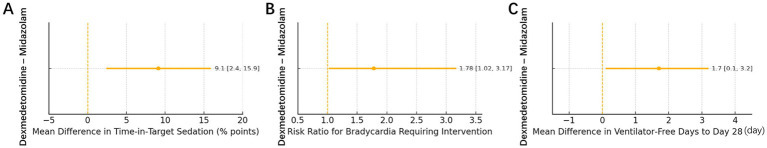
**(A)** Sedation quality. Overlap-weighted mean difference in time-in-target sedation (percentage points), dexmedetomidine minus midazolam: +9.1 [2.4–15.9]. **(B)** Safety signal. Overlap-weighted risk ratio for bradycardia requiring intervention, dexmedetomidine vs. midazolam: 1.78 [1.02–3.17]; vertical line at RR = 1.0. **(C)** Patient-centered outcome. Overlap-weighted mean difference in ventilator-free days up to day 28 (days), dexmedetomidine minus midazolam: +1.7 [0.1–3.2]; vertical line at MD = 0.

Relative to midazolam, dexmedetomidine produced a greater proportion of time spent in target sedation, with a weighted mean difference of +9.1 percentage points (95% CI + 2.4 to +15.9; *p* = 0.008; FDR q = 0.024). Bradycardia requiring intervention occurred more frequently with dexmedetomidine than with midazolam (unweighted 10.5% vs. 5.8%; adjusted RR 1.78, 95% CI 1.02–3.17; *p* = 0.040; FDR q = 0.040) (Table 5 and Figure 3). Hypotension requiring intervention occurred in 22 (13.6%), 30 (15.6%), and 9 (12.5%) patients, respectively, and hypertension requiring treatment occurred in 12 (7.4%), 16 (8.3%), and 5 (6.9%) patients in the dexmedetomidine, midazolam, and remimazolam groups, respectively; overlap-weighted comparisons for dexmedetomidine versus midazolam were not statistically significant (hypotension RR 0.91, 95% CI 0.63–1.32; *p* = 0.620; hypertension RR 0.89, 95% CI 0.52–1.52; *p* = 0.670) (Table 4). The ICU length of stay and 28-day mortality rates were similar across groups, and the overall 28-day mortality rate was 11.3% (Table **6**).

**Table 5:** Secondary and safety outcomes (overlap-weighted).

Outcome	Contrast	Effect (95% CI)	*p*	FDR q
Time in target sedation, % points	Dex vs. Mid	+9.1 (+2.4 to +15.9)	0.008	0.024
Bradycardia requiring intervention, RR	Dex vs. Mid	1.78 (1.02–3.17)	0.040	0.040
Ventilator-free days up to day 28, mean diff (days)	Dex vs. Mid	+1.7 (+0.1 to +3.2)	0.030	0.040
Hypotension requiring intervention, RR	Dex vs. Mid	0.91 (0.63–1.32)	0.620	—
Hypertension requiring treatment, RR	Dex vs. Mid	0.89 (0.52–1.52)	0.670	—

Hypotension and hypertension analyses were exploratory and not included in the prespecified FDR family.

**Table 6:** ICU length of stay and 28-day mortality.

Outcome	Dexmedetomidine (*n* = 162)	Midazolam (*n* = 192)	Remimazolam (*n* = 72)	Overall (*n* = 426)
ICU length of stay (days), median [IQR]	10 [7–15]	11 [8–16]	10 [7–14]	11 [8–16]
28-day mortality, *n* (%)	18 (11.1%)	23 (12.0%)	7 (9.7%)	48 (11.3%)

### Sensitivity analyses

**Figure 4:**
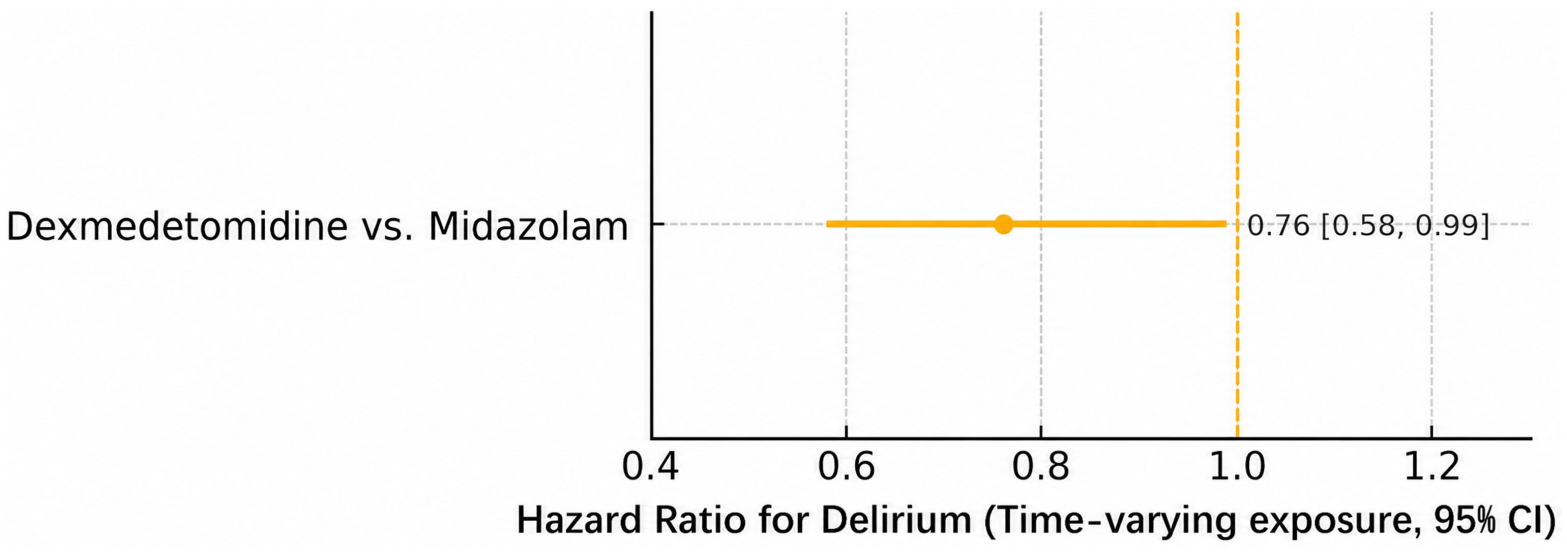
Sensitivity analysis. Time-varying exposure hazard ratio for delirium from ICU admission, with sedative class updated when the predominant continuous sedative changes; follow-up censored at ICU discharge, death, or ICU day 7. Dexmedetomidine vs. midazolam: 0.76 [0.58–0.99]; vertical line at HR = 1.0.

**Figure 5:**
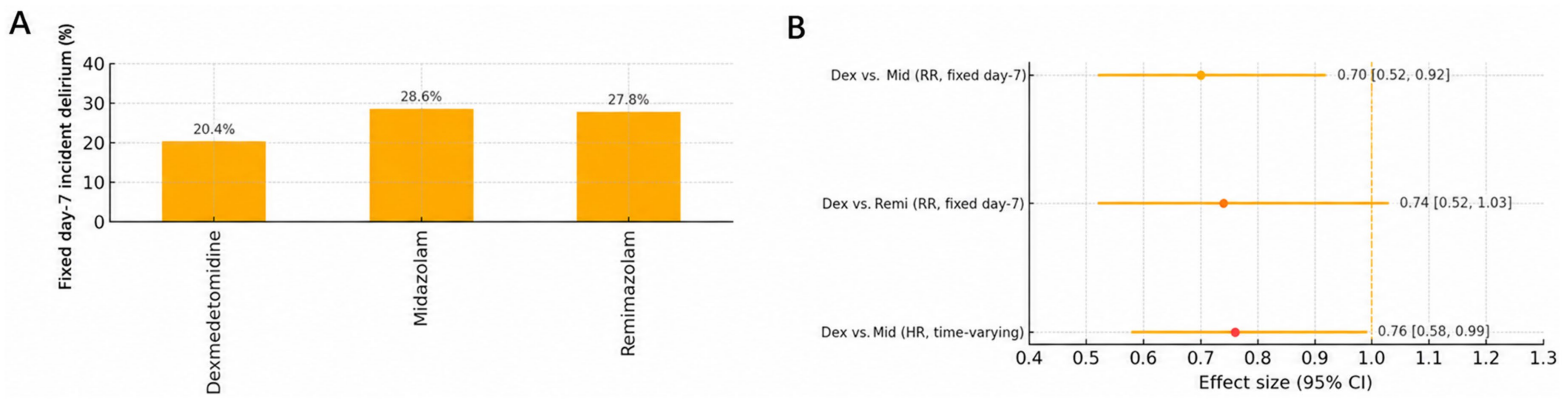
**(A)** Fixed day-7 post-landmark delirium risk (unadjusted). Group-level absolute risks of incident delirium from hour 48 to ICU day 7/discharge; these anchor the fixed-time analysis. **(B)** Effect estimates: fixed day-7 vs. time-to-event. Overlap-weighted risk ratios at a fixed horizon (day 7) are shown alongside a time-varying Cox hazard ratio from ICU admission and a Fine-Gray subdistribution hazard ratio treating ICU discharge and death before delirium as competing events, providing complementary fixed-risk and time-to-event perspectives.

In the prespecified, time-varying sensitivity analysis from ICU admission, which modeled sedative class as a time-updated covariate and included all delirium events, the association remained directionally consistent with that of the primary analysis, with a hazard ratio of 0.76 (95% CI 0.58–0.99; *p* = 0.041) for dexmedetomidine versus midazolam (Figure 4). In an as-treated post-landmark analysis, which censored follow-up at the first switch away from the predominant sedative, the hazard ratio for dexmedetomidine versus midazolam was 0.73 (95% CI 0.54–0.98; *p* = 0.036). In the worst-case analysis classifying patients with ≥2 consecutive unassessable CAM-ICU assessments (SAS < 3) as having delirium, the adjusted RR for dexmedetomidine versus midazolam was 0.74 (95% CI 0.58–0.95; *p* = 0.018). Competing-risk analyses treating ICU discharge and death before delirium as competing events yielded directionally consistent estimates (Figure 5).

## Discussion

In this real-world ICU cohort using standardized delirium surveillance and a prespecified 48-h landmark to align exposure with risk, dexmedetomidine-predominant sedation was associated with a lower adjusted risk of ICU delirium, greater time in target sedation, and more ventilator-free days than midazolam, whereas mortality did not meaningfully differ. Remimazolam estimates were imprecise and not statistically different from those of either comparator. These findings are hypothesis-generating and highlight the need for larger, multicenter studies to confirm comparative effectiveness and safety, particularly to clarify the role of remimazolam.

Our findings of lower incident ICU delirium with dexmedetomidine versus midazolam after the 48-h landmark, together with more time in target sedation, are biologically consistent with dexmedetomidine’s α2-agonist action in the locus coeruleus, which produces cooperative, arousable, non-GABAergic sedation that permits engagement and cognitive testing rather than amnestic oversedation (17, 18). Dexmedetomidine also better preserves elements of sleep architecture relevant to cognition. Quantitative ICU EEG shows more NREM-like patterns, yet persistent vulnerability to sleep fragmentation, underscoring why pharmacology must be paired with sleep-protective care bundles (19). These mechanisms align with the contemporary guideline emphasis on light, goal-directed sedation and structured delirium surveillance in ICU delirium (8).

Placed in the context of randomized and pooled evidence, our effect estimates are directionally concordant with trials showing less delirium and earlier extubation with dexmedetomidine compared with benzodiazepines in mechanically ventilated adults and with non-inferior sedation quality relative to propofol or midazolam (20–22). A large meta-analysis of RCTs similarly reported lower risk of delirium with dexmedetomidine versus other sedatives, albeit with more bradycardia (3). Observational data also associated GABA-A benzodiazepines with a higher risk of delirium than propofol in the ICU, which reinforces the biologic plausibility that GABAergic amnesia and sleep disruption can exacerbate acute brain dysfunction in ICU delirium (23, 24). Consistent with this body of evidence, we observed more ventilator-free days with dexmedetomidine than with midazolam and imprecise, non-significant estimates for remimazolam, which is mechanistically GABA-A but pharmacokinetically short-acting, and it still has limited direct ICU delirium data (3, 25, 26).

The observed safety trade-off of higher bradycardia requiring intervention with dexmedetomidine is pharmacologically expected from central sympatholysis and vagotonia and mirrors RCT and meta-analytic signals (3, 20). In contrast, hypotension and hypertension requiring intervention were not statistically different across regimens in our cohort, although the estimates were imprecise, and these safety analyses were not powered for rare events. Because misclassification can bias delirium ascertainment in deeply sedated patients, our protocolized, “sedation-gated” CAM-ICU approach follows foundational validation work and current guidance and supports the internal validity of our ICU delirium outcome (9, 15, 27). We additionally quantified unassessable CAM-ICU assessments due to deep sedation and performed a worst-case sensitivity analysis, with directionally consistent results. Overall, the convergence of mechanisms, trials, and guidelines strengthens the interpretation that dexmedetomidine’s arousable, non-GABAergic sedation may reduce the risk of delirium compared with benzodiazepines while necessitating vigilant heart-rate monitoring and judicious dosing. However, emerging remimazolam data warrant multicenter confirmation before firm inferences regarding ICU delirium can be made (3, 8, 25, 26).

Limitations of our study include its single-center design, the possibility of residual or unmeasured confounding factors (e.g., unrecorded neurological vulnerability), potential exposure switching after 48 h (which was addressed but not eliminated by the sensitivity analyses), imprecision due to the smaller remimazolam group, and the risk that delirium ascertainment is hampered by deep sedation despite standardized, sedation-gated assessments. The cohort included patients with heterogeneous admission diagnoses, and we did not collect validated baseline delirium-risk prediction scores (e.g., PRE-DELIRIC), leaving potential residual confounding by underlying delirium susceptibility (28). The landmark design necessarily excluded patients with early delirium and those who died or were discharged before 48 h, which may introduce selection bias and limit the generalizability to longer-stay ICU survivors. Although time-varying and as-treated analyses were directionally consistent, we did not apply marginal structural models to fully address time-varying confounding by indication, and safety event analyses were underpowered for rare outcomes. Local sedation protocols, nursing staffing, and drug availability (including remimazolam) may also limit external generalizability. Accordingly, sedation selection should balance the potential benefits of preventing delirium with hemodynamic tolerability and patient-specific factors. The results for remimazolam were imprecise and not statistically different from those for dexmedetomidine, underscoring the need for adequately powered multicenter studies to clarify its comparative effectiveness and safety.

Taken together, our observational data suggest that dexmedetomidine-predominant sedation is associated with a lower post-landmark risk of ICU delirium and improved sedation quality compared with midazolam, with expected bradycardia that did not translate to excess mortality, while remimazolam showed intermediate, imprecise estimates. These findings should be interpreted as associations in an equipoise-weighted population from a single center and thus require multicenter confirmation and randomized trials, particularly to clarify the role of remimazolam in ICU delirium prevention.

## Data Availability

The raw data supporting the conclusions of this article will be made available by the authors, without undue reservation.
